# Supplementary material for: Mosquitocidal Activity of the Methanolic Extract of Annickia chlorantha and Its Isolated Compounds against Culex pipiens, and Their Impact on the Non-Target Organism Zebrafish, Danio rerio
Source: Insects. 2022 Jul 27;13(8):676. doi: 10.3390/insects13080676 (PMC9409109; doi:10.3390/insects13080676)
Supplement: Supplementary file 1 [file insects-13-00676-s001.zip › insects-1801527-supplementary.pdf]

Table S1: Larvicidal activity of palmatine fraction against the mosquito vector, *Culex pipiens.*

Concentrations (µg/ml)	*n*	Larval mortality % ± SE	Regression equation	LC_{50} (LCL–UCL) (µg/ml)	LC_{90} (LCL–UCL) (µg/ml)	Statistic summary
Control	75	0.0 ± 0.0a	Y = 1.3293X - 5.1626	33.392 (27.366 – 40.343)	81.522 (63.405 – 122.239)	*d.f.*= 5, *P*< 0.001, χ^2= 7.121
10	75	9.33 ± 1.33b				
20	75	21.33 ± 1.33c				
40	75	42.67 ± 1.33d				
60	75	81.33 ± 2.67e				
80	75	98.67 ±1.33f				

Larval mortalities are presented as Mean±SE of three replicates. Means with different letters are significantly differentat (P<0.05). (LC_{50}) concentration that kills 50% of population, (LC_{90}) concentration that kills 90% of population, (LCL) lower confidence limit, (UCL) upper confidence limit, (DF) degree of freedom, (χ^2) Chi-square, *n*= sample size.

Table S2: Larvicidal activity of jatrorrhizine fraction against the mosquito vector, *Culex pipiens*.

Concentrations (µg/ml)	*n*	Larval mortality % ± SE	Regression equation	LC_{50} (LCL–UCL) (µg/ml)	LC_{90} (LCL–UCL) (µg/ml)	Statistic summary
Control	75	0.0 ± 0.0a	Y = 0.5161X - 6.7236	91.343 (74.861 – 110.929)	228.135 (174.962 – 354.875)	*d.f.*= 5, *P*< 0.001, χ^2 = 5.140
25	75	6.67± 1.33ab				
50	75	20.0 ± 4.0b				
100	75	42.67 ± 4.81c				
150	75	70.67 ± 3.53d				
200	75	97.33 ± 1.33e				

See footnote Table 1.

Table S3: Larvicidal activity of columbamine fraction against the mosquito vector, *Culex pipiens*.

Concentrations (µg/ml)	*n*	Larval mortality % ± SE	Regression equation	LC_{50} (LCL–UCL) (µg/ml)	LC_{90} (LCL–UCL) (µg/ml)	Statistic summary
Control	75	0.0 ± 0.0a	Y = 1.1267X - 23.867	61.440 (53.260 – 70.980)	119.542 (97.470 – 171.995)	*d.f.*= 5, *P*< 0.001, $\chi^2 = 6.860$
20	75	5.33 ± 2.67ab				
40	75	18.67 ± 3.51b				
60	75	34.67 ± 3.51c				
80	75	65.33 ± 4.81d				
100	75	94.67 ± 1.33e				

See footnote Table 1.

Table S4: Larvicidal activity of *Beta*-sitosterol fraction against the mosquito vector, *Culex pipiens*.

Concentrations (μg/ml)	*n*	Larval mortality % ± SE	Regression equation	LC_{50} (LCL–UCL) (μg/ml)	LC_{90} (LCL–UCL) (μg/ml)	Statistic summary
Control	75	0.0 ± 0.0a	Y = 0.456X - 14.267	123.236 (52.365 – 210.466)	254.709 (166.189 – 4711.963)	*d. f.*= 5, *P*< 0.001, χ^2 = 8.006
50	75	12.0 ± 0.0b				
100	75	29.33 ± 2.67c				
150	75	48.0 ± 2.31d				
200	75	81.33 ± 1.33e				
250	75	100.0 ± 0.0f				

See footnote Table 1.

Table S5: Larvicidal activity of the methanolic extract of *Annickia chlorantha* against the mosquito vector, *Culex pipiens.*

Concentrations (µg/ml)	*n*	Larval mortality % ± SE	Regression equation	LC_{50} (LCL–UCL) (µg/ml)	LC_{90} (LCL–UCL) (µg/ml)	Statistic summary
Control	75	0.0 ± 0.0a	Y = 0.2499X - 1.0163	162.630 (144.472 – 182.347)	433.95 (365.123 – 546.16)	*d.f.*= 5, *P*< 0.001, χ^2 = 19.459
50	75	9.33± 2.67b				
100	75	28.0 ± 2.31c				
200	75	45.33 ± 3.53d				
300	75	77.34 ± 3.53e				
400	75	97.33 ± 1.33f				

See footnote Table 1.
